# Unfolding the Antibacterial Activity and Acetylcholinesterase Inhibition Potential of Benzofuran-Triazole Hybrids: Synthesis, Antibacterial, Acetylcholinesterase Inhibition, and Molecular Docking Studies

**DOI:** 10.3390/molecules28166007

**Published:** 2023-08-10

**Authors:** Sadaf Saeed, Ameer Fawad Zahoor, Shagufta Kamal, Zohaib Raza, Mashooq Ahmad Bhat

**Affiliations:** 1 Department of Chemistry, Government College University Faisalabad, Faisalabad 38000, Pakistan; sadafsaeed932@gmail.com; 2 Department of Biochemistry, Government College University Faisalabad, Faisalabad 38000, Pakistan; shaguftakamal81@gmail.com; 3 Department of Chemistry, School of Physical Sciences, University of Adelaide, Adelaide, SA 5000, Australia; zohaib.raza@adelaide.edu.au; 4 Department of Pharmaceutical Chemistry, College of Pharmacy, King Saud University, Riyadh 11451, Saudi Arabia

**Keywords:** benzofuran-triazole, microwave, acetylcholinesterase inhibitors, molecular docking, structure-activity relationship, antibacterial activity

## Abstract

In this study, a series of novel benzofuran-based 1,2,4-triazole derivatives (**10a–e**) were synthesized and evaluated for their inhibitory potential against acetylcholinesterase (AChE) and bacterial strains (*E. coli* and *B. subtilis*). Preliminary results revealed that almost all assayed compounds displayed promising efficacy against AChE, while compound **10d** was found to be a highly potent inhibitor of AChE. Similarly, these 5-bromobenzofuran-triazoles **10a**–**e** were screened against *B. subtilis* QB-928 and *E. coli* AB-274 to evaluate their antibacterial potential in comparison to the standard antibacterial drug penicillin. Compound **10b** was found to be the most active among all screened scaffolds, with an MIC value of 1.25 ± 0.60 µg/mL against *B. subtilis*, having comparable therapeutic efficacy to the standard drug penicillin (1 ± 1.50 µg/mL). Compound **10a** displayed excellent antibacterial therapeutic efficacy against the *E. coli* strain with comparable MIC of 1.80 ± 0.25 µg/mL to that of the commercial drug penicillin (2.4 ± 1.00 µg/mL). Both the benzofuran-triazole molecules **10a** and **10b** showed a larger zone of inhibition. Moreover, IFD simulation highlighted compound **10d** as a novel lead anticholinesterase scaffold conforming to block entrance, limiting the swinging gate, and disrupting the catalytic triad of AChE, and further supported its significant AChE inhibition with an IC_50_ value of 0.55 ± 1.00 µM. Therefore, compound **10d** might be a promising candidate for further development in Alzheimer’s disease treatment, and compounds **10a** and **10b** may be lead antibacterial agents.

## 1. Introduction

Alzheimer’s disease (AD) is a multifactorial and age-related neurological disorder that often leads to cognitive dysfunction accompanied by a progressive memory loss, disorientation, decline in language, and behavioral abnormalities [1,2,3]. The World Health Organization (WHO) estimated that more than 50% of people are currently living with AD in the developing world and this may increase to 70% by 2025 [4]. Although several factors have been attributed to AD over the years, its etiology is still unknown [5]. As a number of hypotheses have been reported, including the tau hypothesis, oxidative stress, the cholinergic hypothesis, neuroinflammation, and the amyloid hypothesis, several drugs that target these hypotheses have been studied for the treatment of AD [6]. The cholinergic hypothesis suggests that the decline in cholinergic neurons in AD results in a decline of acetylcholine (ACh) in specific brain regions that regulate memory and cognition functions [7]. Consequently, one of the most promising strategies adopted for AD treatment is to enhance the acetylcholine levels in the brain by targeting the AChE enzyme [8].

**Figure 1:**
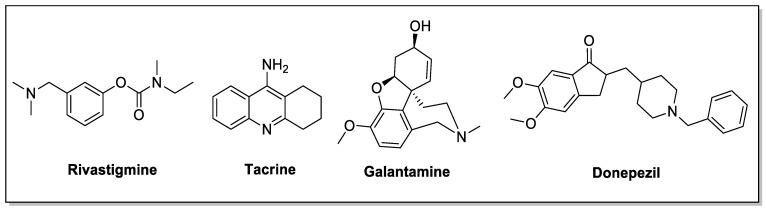
Approved AChE inhibitors.

Acetylcholinesterase (AChE) is a neuro-enzyme that terminates impulse transmission at cholinergic synapses through the fast hydrolysis of acetylcholine (ACh) into choline and acetate [9,10,11]. Therefore, inhibition of AChE is the most effective therapeutic approach to restore ACh concentrations in the brain and slows down Alzheimer’s disease (AD) symptoms [12,13]. The AChE inhibitors reduce the acetylcholine (ACh) hydrolysis; consequently, ACh levels increase at the synaptic cleft, which can stimulate cholinergic receptors and further promote memory function [14]. According to these findings, the U.S. FDA has approved four AChE inhibitors: rivastigmine, tacrine, galantamine, and donepezil [15,16] for the treatment of dementia, delirium, traumatic brain injury, and AD (Figure 1). However, these drugs have low bioavailability and are associated with a variety of side effects such as gastrointestinal upsets, syncope, increased risk of bradycardia, and hepatotoxicity [17]. Therefore, development of novel AChE inhibitors with lesser side effects and better efficacy is necessary. This has attracted the attention of medicinal researchers to develop novel AChE inhibitors. 

**Figure 2:**
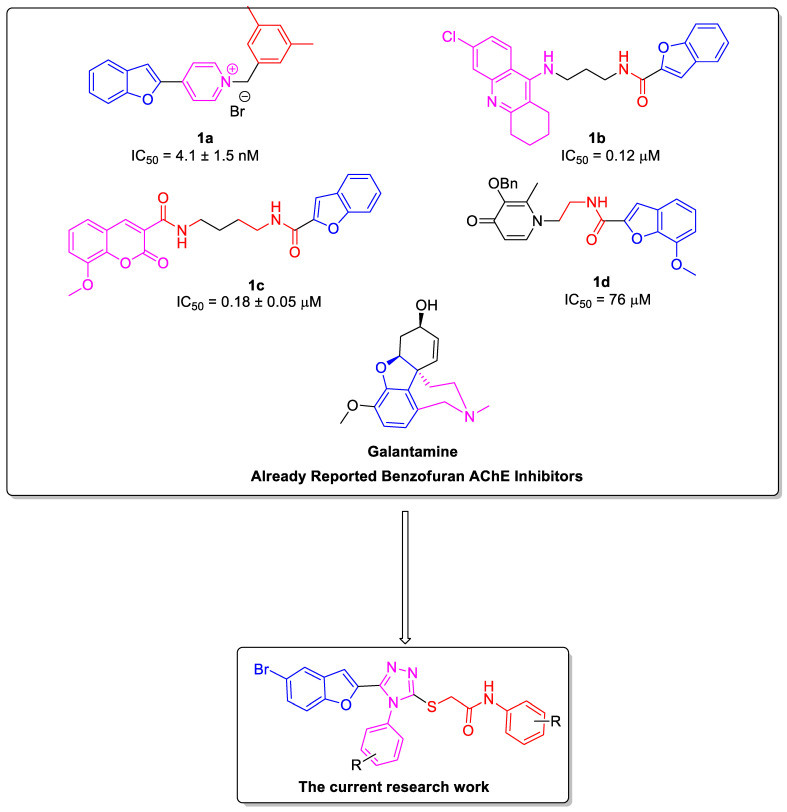
Rational design of benzofuran-triazoles as anti-AChE agents.

The heterocyclic scaffolds such as benzofuran [18,19], pyrazole [20], lamivudine [21], quinoxaline-sulfonamide [22], oxadiazole [23,24,25,26], triazole [27,28], phenyl-piperazine-based carbodithioates [29,30,31], and thiadiazole [32,33] have drawn significant attraction in the area of medicinal chemistry due to their remarkable pharmacological and biological activities [34,35]. In particular, benzofuran has attracted researchers because it is so frequent in natural products [36]. Benzofuran scaffolds exhibit numerous biological properties such as antimicrobial, antidiabetic, analgesic, antitumor, kinase inhibiting and anti-Alzheimer’s properties [37,38]. Recently, heterocyclic scaffolds containing the benzofuran ring have been reported as potent AChE inhibitors, such as benzofuran-based benzylpyridinium **1a**, tacrine-derived benzofuran **1b**, coumarin-linked benzofuran **1c**, and hydroxypyridinone-containing benzofuran **1d** [39,40,41,42] (Figure 2).

There is significant evidence that benzofuran can be linked to a variety of other anti-AChE compounds such as triazoles to enhance their anti-AChE potential [43,44]. 1,2,4-Triazole is a prominent fragment present in various drugs [45] and its analogues are known to possess remarkable pharmacological activities, such as antibacterial, anticonvulsant, analgesic, antiviral, anti-inflammatory, antidepressant, antitubercular, antifungal, hypoglycemic, sedative, hypnotic, antiparasitic, insecticidal, and cholinesterase inhibiting properties [46,47,48]. 

**Figure 3:**
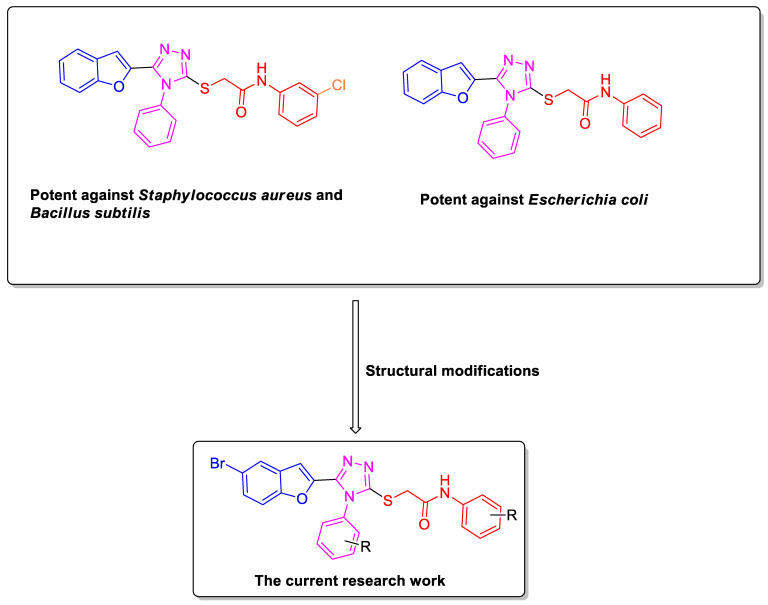
Rational design of benzofuran-triazoles as antibacterial agents.

Similarly, 1,2,4-triazole-derived compounds are also reported as powerful pharmacophores owing to their chemotherapeutic potential, including their antibacterial activity, towards drug-resistant pathogens [49]. Our research group has already identified benzofuran-oxadiazole and benzofuran-triazole (Figure 3) as promising antibacterial agents [50]. Furthermore, a number of 1.2.4-triazole derived drugs including fluconazole and ribavirin are already employed in clinics to treat various diseases. Consequently, 1,2,4-triazole derivatives play an important role in new drug development [51].

Ultrasound and microwave irradiation methodologies have gained attention in organic synthesis over conventional methods. The benefits of these synthetic methodologies are simplicity, shorter reaction time, and products formed with higher yield, which make these techniques more effective and eco-friendlier [52].

Based on these considerations, ultrasound irradiation and conventional and microwave-assisted protocols were used to synthesize hybrid compounds incorporating 1,2,4-triazole and benzofuran moieties, which are novel AChE inhibitors with high potency. The synthesized compounds were also studied for their antibacterial potential.

## 2. Results and Discussion

### 2.1. Chemistry

**Scheme 1:**
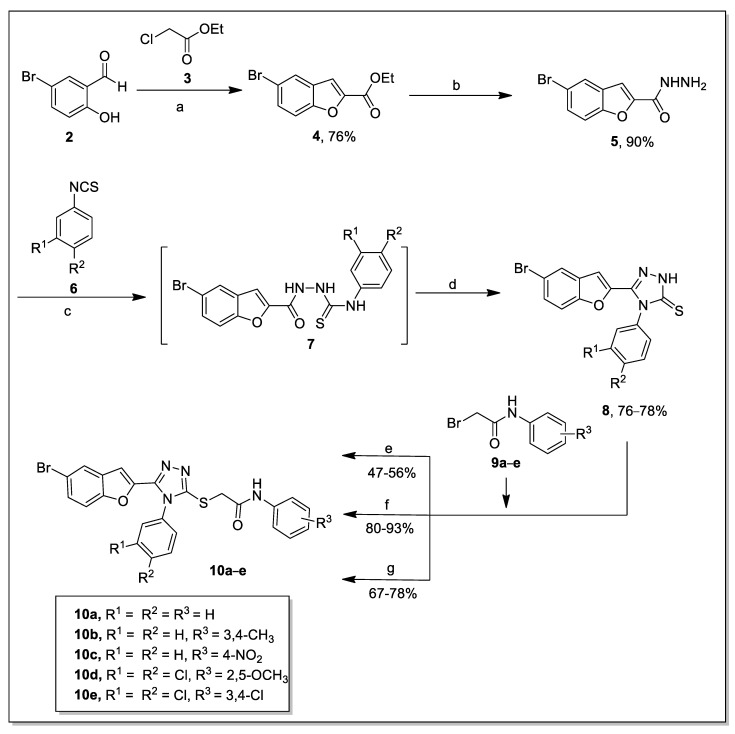
Synthetic pathway for the synthesis of benzofuran-based 1,2,4-triazole derivatives **10a**–**e**. Reagents and conditions: (a) K_2_CO_3_, DMF, 92–94 °C, 4 h. (b) NH_2_NH_2_^.^ H_2_O, 25 °C, overnight. (c) ethanol, reflux, 2 h. (d) Aq KOH, reflux, 4 h. (e) dimethylformamide, lithium hydride, stirring at 25 °C, 15–24 h. (f) dimethylformamide, lithium hydride, microwaves, 60–75 s. (g) triethylamine, ethanol, 15–30 min, ultrasonic irradiation.

The synthetic approach of benzofuran-based 1,2,4-triazole derivatives is illustrated in Scheme 1. 5-Bromo-2-hydroxybenzaldehyde **2** was reacted with ethyl chloroacetate **3** in the presence of K_2_CO_3_ to afford ethyl benzofuran-2-carboxylate **4** (76%) [50]. Hydrazinolysis of ester **4** with hydrazine monohydrate yielded 5-bromobenzofuran-2-carbohydrazide **5** as a white solid in a 90% yield [53]. Furthermore, synthesis of thiosemicarbazide (intermediate) **7** was achieved by the treatment of hydrazide **5** with isothiocyanate **6** in ethanol. Subsequently, cyclocondensation of key intermediate **7** in aqueous alkaline medium at reflux furnished the corresponding 1,2,4-triazole-3-thione **8** (76–78%) [26]. Finally, the synthesis of benzofuran-triazole derivatives **10a**–**e** was smoothly accomplished by reaction of corresponding triazole **8** containing a benzofuran moiety with various 2-bromo-*N*-phenylacetamides **9a**–**e** via three steps, e–g. This conversion was carried out by employing conventional heating, MW [54], and ultrasound irradiation [55] techniques. Upon comparison with conventional heating, it was determined that ultrasound irradiation increased the yields from 47–56% to 67–78% and decreased the reaction time from 15–24 h to 15–30 min. Meanwhile, the microwave-assisted synthetic strategy afforded a maximum yield of products of 80–93% in 60–75 s. The results listed in Table 1 demonstrate that microwave-assisted methodology is preferable because of its shorter reaction time and higher yield.

### 2.2. Spectral Interpretation of Compound (***10d***)

**Figure 4:**
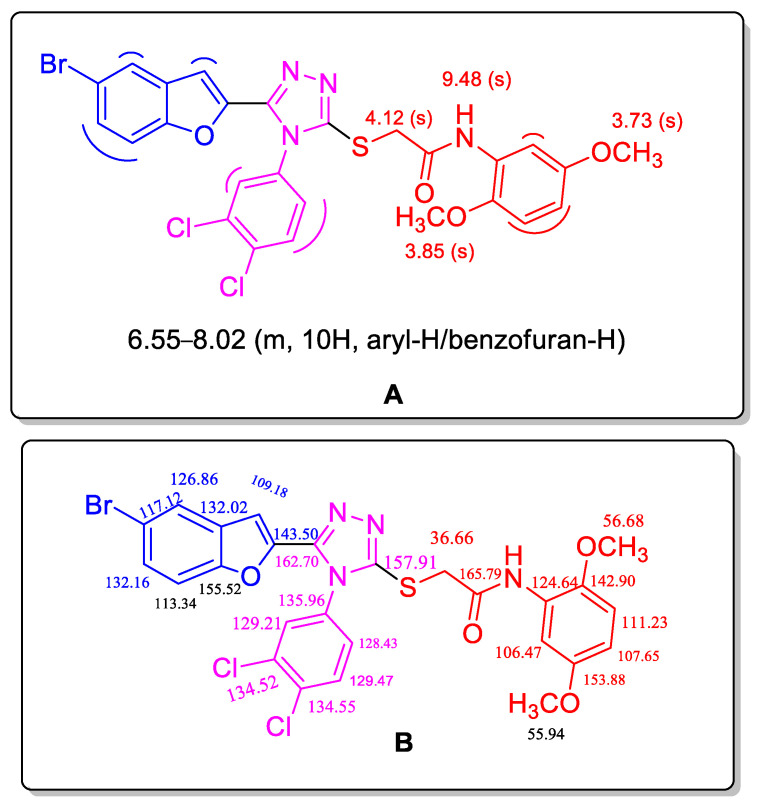
^1^HNMR (**A**) and ^13^C NMR (**B**) of the compound **10d**.

Structural elucidation of compound **10d** was confirmed by ^1^HNMR and ^13^CNMR spectroscopy. In the ^1^HNMR spectrum, signals for methylene (linker) and NH of the acetamide appeared as singlets at δ 4.12 and δ 9.48, respectively. The presence of two methoxy groups at the anilide aryl ring was confirmed by the appearance of two singlets at 3.73 and 3.85. Multiplet signal at δ 6.55–8.02 appeared for aryl and benzofuran rings (Figure 4A). 

The ^13^C-NMR spectrum also supported the carbon framework of **10d**. All carbons displayed signals in the spectrum, and two resonating signals at δ 165.79 for the carbonyl group and at δ 36.66 for the methylene certified the presence of the acetamide group. Two downfield signals confirmed the formation of 1,2,4-triazole rings, one at δ 157.91 and one at δ 162.70. The carbons of the substituted aryl ring attached to triazole depicted their signals at δ 134.52 and δ 134.55 for C-Cl and δ 135.95 for C-N, whereas the remaining signals of three carbons were seen at δ 128.43, δ 129.21, and δ 129.47. Signals indicating the formation of benzofuran rings appeared at δ 109.18, δ 113.34, δ 117.12, δ 126.86, δ 132.02, δ 132.16, δ 143.50, and δ 155.52. The 2,5-dimethoxyphenyl ring attached to the acetamide functional group displayed two signals at δ 142.90 and δ 153.88 for C-OCH_3_ and at δ 124.64 for C-N, while other methine signals were observed at δ 106.47, δ 107.65, and δ 111.23. Two methoxy substituents at the aryl ring depicted signals on the right side of the spectrum at δ 55.94 and δ 56.68. All other synthetic compounds in the series (**10a**–**e**) were characterized structurally using a similar approach (Figure 4B). 

### 2.3. AChE Inhibitory Activity

The in vitro inhibitory activity of benzofuran-linked 1,2,4-triazoles toward AChE was evaluated by employing Ellman’s method [56,57] and using eserine as the reference drug. Results indicated that all synthesized compounds were less potent as compared to eserine. The obtained IC_50_ values for AChE inhibition are depicted in Table 2. The percentage inhibition ranged from 51.23 ± 1.24 mM to 81.45 ± 0.50 mM and their IC_50_ values from 2.28 ± 1.75 μM to 0.55 ± 1.00 μM. Among those tested, compound **10d** displayed strong AChE inhibitory activity, with an IC_50_ value of 0.55 ± 1.00 μM. Meanwhile, compound **10a** also proved to be a good inhibitor with a cell viability value, i.e., 0.88 ± 0.50 μM. Compounds **10b**, **10c**, and **10e** were found to have moderate inhibitory activities with IC_50_ values of 1.50 ± 1.00 μM, 2.28 ± 1.75 μM, and 1.98 ± 0.25 μM, respectively.

### 2.4. Structure–Activity Relationship of the Potent AChE Inhibitors

**Figure 5:**
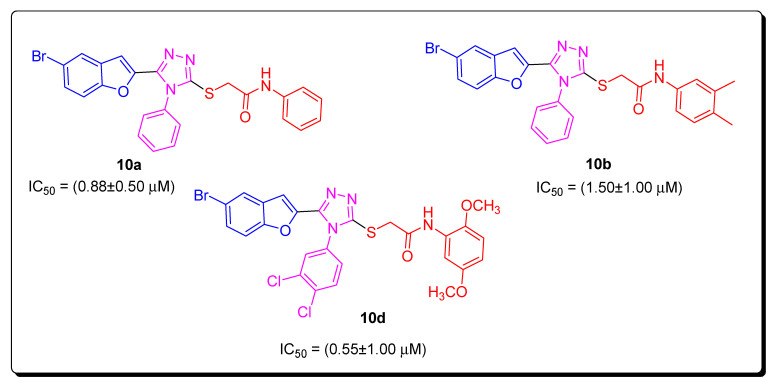
SAR of the bioactive **10a**, **10b**, and **10d** AChE inhibitors.

The SAR (structure–activity relationship) for all target compounds regarding AChE inhibitory activity was analyzed on the basis of the position and nature of substituents on the aryl ring of *N*-phenylacetamide and on the phenyl ring linked to the 1,2,4-triazole ring. An investigation of the compounds’ structure and activities revealed that introduction of electron-donating groups slightly enhanced the AChE inhibitory activity. For instance, compound **10a** possessing unsubstituted phenyl rings was the second most potent derivative of the series, with an IC_50_ value of 0.88 ± 0.50 μM. In this series, 3,4-dimethylphenyl derivative **10b** (cell viability = 1.50 ± 1.00 μM) was less active than **10a**. Thus, introduction of nitro-substitution at the para position of the *N*-phenylacetamide, as in compound **10c** (cell viability = 2.28 ± 1.75 μM), led to a further decrease in activity. Based on these findings, more modifications could be undertaken on the aryl ring linked to the 1,2,4-triazole scaffold. Therefore, by positioning the chloro group into the R^1^ and R^2^ position, the influence of R groups on AChE inhibitory activity could be further explored. Compound **10d** bearing the 2,5-dimethoxyphenyl moiety and exhibiting less cell viability (0.55 ± 1.00 μM) was found to be the most active anti-AChE agent among all the synthetic derivatives. Moreover, changing the position and nature of the substituent in the acetamide ring from 2,5-dimethoxyphenyl to 3,4-dichlorophenyl produced **10e** (1.98 ± 0.25 μM) and led to a decrease in inhibitory activity (Figure 5).

### 2.5. Antibacterial Activity

The evaluation of in vitro antibacterial activity of synthesized compounds was performed against two bacterial strains, namely Gram +ve *B. subtilis* QB-928 and Gram −ve *E. coli* AB-274 using the disc diffusion method [58,59]. The results depicted in Table 3 indicate that five compounds **10a**–**e** exhibited moderate to excellent antibacterial activities as compared to the standard drug. In particular, compound **10b** showed potent activity against *B. subtilis*, with a diameter of the zone of inhibition of 42 mm and MIC value of 1.25 μg/mL, when compared with the standard drug penicillin, which has an inhibition value of only 18 mm and an MIC value of 1 μg/mL. In addition, compound **10a** demonstrated the most promising activity against *E. coli* (38 mm), with a MIC value of 1.80 μg/mL. Compound **10c** exhibited moderate antibacterial activity, that is, 28 mm. The antibacterial effect of **10e** against *E. coli* was found to be similar in activity to that of the standard antibiotic, that is, 24 mm. Moderate activity values of 12 mm and 11.5 mm were shown by the two derivatives of **10d** and **10b** against *E. coli*, respectively, when used against Gram-positive bacteria (*B. subtilis*); compounds **10a** and **10c** produced no detectable results. 

Among all the benzofuran–triazole derivatives (**10a**–**e**), compound **10b** with CH_3_ (electron-donating) substituents showed the highest activity against *B. subtilis* (42 mm) but was less active against *E. coli* (11.5 mm). The second most active compound in the series was **10a**, which has unsubstituted phenyl rings. The presence of electron-withdrawing substituent NO_2_ at the para position on the anilide ring decreased the antibacterial activity of compound **10c**. Introducing a chloro group at both the meta and the para positions of the anilide ring, **10e** exhibited activity similar to that of standard penicillin against *E. coli* (24 mm) but it was relatively less effective towards *B. subtilis* (8 mm). Placing it with methoxy substituents at *N*-phenyl acetamide resulted in **10d**, which was less active towards *B. subtilis* (11 mm) and *E. coli* (12 mm). 

### 2.6. In Silico Modeling of the Most Potent Compound (***10d***)

The anticholinesterase activity of compound **10d** was further modeled in IFD to simulate its interactions and binding behavior with the AChE active site and provided insights into its potential mechanism of action responsible for AChE inhibition. The docking protocol was validated by cognate re-docking, where it was found to reproduce the co-crystalized binding pose with a 1.5 RMSD, as depicted in Supplementary Figure S11.

Since poses of docked compounds were ranked based on their free energy to stabilize their conformation within the binding pocket, the pose with lowest binding energy (∆G) was chosen as a representative conformation and highlighted its potential ∆G as a function of its binding affinity. Galantamine was found to stabilize its conformation with −7.07 Kcal/mol of ∆G and acted as a reference threshold for IFD (Table 4). Interestingly, **10d** crossed the standard threshold and exhibited the superior binding affinity with −9.34 Kcal/mol of ∆G, which may imply its potential AChE inhibitory potential. 

**Figure 6:**
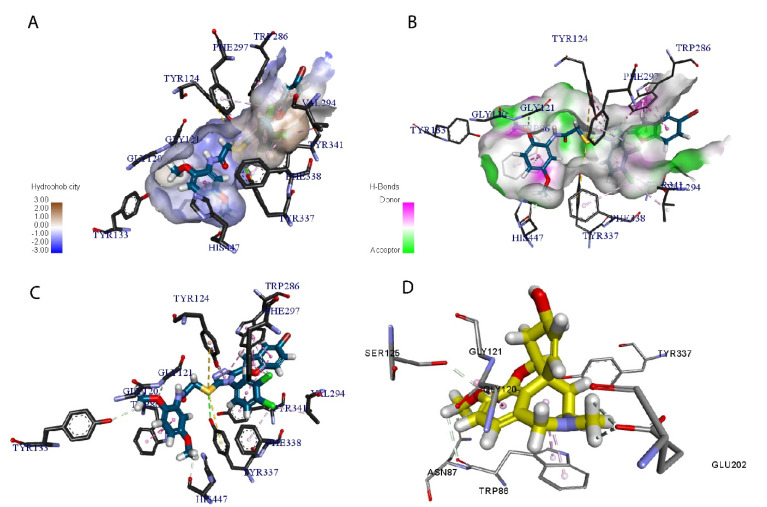
Conformational analysis of ligands within AChE active sites; simulated best binding mode of **10d** (**A**–**C**) and galantamine (**D**) in the 3D space of the AChE binding pocket.

The poses with the lowest binding energy were further modeled for ligand–receptor interactions and behavior within various important sites of AChE active sites where galantamine and compound **10d** were found to efficiently penetrate up to its catalytic site (Figure 6). The AChE active site’s entrance is a narrow gorge extensively attached with residues having aromatic side chains with low affinity toward acetylcholine, thus creating an aromatic guidance phenomenon for acetylcholine diffusion into an AChE catalytic triad (GLU-SER-HIS). The aromatic channel within the active site gorge of AChE also renders a narrower entrance and provides selectivity for compounds with a suitable molecular size or steric effect owing to their flexibility of aromatic side chains. Galantamine exhibited a conformation that effectively crossed this aromatic channel and diffused all the way towards the catalytic triad (Figure 6D). Notably, the conformational analysis of **10d** revealed its efficient diffusion or passage through this narrow hydrophobic aromatic channel; thus, it was absorbed and stabilized into the hydrophilic pocket of the active site, with potential sites for hydrogen bonding where it may disrupt the catalytic triad of AChE (Figure 6A–C).

**Figure 7:**
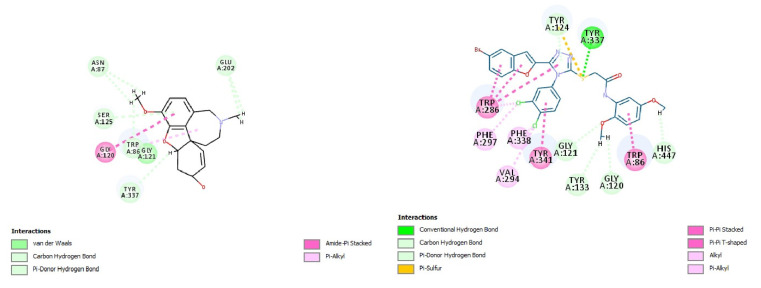
Interaction behavior of galantamine (**left**) and **10d** (**right**) within the AChE active site; ligand interactions in a 4D perspective with key residues depicted as a ball and stick model colored in relation to the nature of bonding.

Galantamine was observed to stabilize its conformation by undergoing H-bonding with ASN87, SER125, TRP86, GLY121, TYR337, GLU202, and hydrophobic interactions with GLY120 and provided insights into the interaction profiles of potential inhibitors (Figure 7). Interestingly, **10d** was found to inhibit the narrow active site gorge or channel by forming hydrophobic interactions with TRP286, PHE228, PHE297, and VAL294, and stabilized its binding pose within this hydrophobic cavity and blocked the entrance to the AChE active site. Furthermore, **10d** was also found to inhibit the swinging gate (i.e., TYR337) of the catalytic site by forming H-bonds with it and its corresponding residue (TYR133), thus supporting its anchorage throughout the active site. It also blocked the oxyanion hole of the catalytic site by H-bonding with GLY120 and GLY121 to further limit the catalytic process of AChE. However, upon its stabilization of conformation, **10d** disrupts the catalytic triad by forming an H-bond with HIS447, which may ultimately lead to blockade of AChE catalysis. 

Induced Fit docking (IFD) is a powerful tool used to simulate the binding of ligands to their receptors which considers the flexible spatial alignment of side chains of residues with the binding pocket upon ligand binding, thus simulating the accurate binding mode of the ligand and its interactions or behavior within the binding pocket. Herein, **10d** was found to exhibit superior binding affinity, as highlighted with the lowest binding energy required for its conformation to stabilize within the binding pocket. This affinity was further highlighted considering its important interactions to stabilize its conformation where it was found to block the AChE active site entrance gorge, interact with the swinging gate to further control the stabilization of the pose, and disrupt the catalytic triad of AChE. Therefore, IFD simulation further supported the experimental studies to identify **10d** as the potential lead scaffold for AChE inhibition and provided important insights to strengthen the ground to optimize and develop novel anticholinesterase lead candidates based on this scaffold in therapeutics of Alzheimer’s disease.

## 3. Experimental

Ultrasonic irradiation and microwave-assisted experiments were carried out, respectively, in an ultrasonic cleaner bath (Model 1510) with a frequency of 47 kHz and power consumption of 115 v, and with a microwave apparatus (Model EA-180M, 1150 watt) at a frequency of 2450 Hz. All the chemicals, starting materials, and solvents employed in this synthetic strategy were of analytical grade and obtained from Merck or Sigma Aldrich and used without further purification. For monitoring the reaction progress and purity of compounds, thin layer chromatography was performed using silica gel plates. However, a UV lamp was used to visualize the spots on a TLC plate. Melting points of target analogs were determined using Gallenkamp equipment. Spectral analyses of ^1^H-NMR at 400 MHz (δ = ppm) and ^13^C-NMR at 100 MHz (δ = ppm) were carried out on a Bruker spectrophotometer. 

### 3.1. General Protocol for the Synthesis of Derivatives

#### 3.1.1. Synthesis of Ethyl 5-Bromobenzofuran-2-Carboxylate (**4**)

To a solution of 5-bromo-2-hydroxybenzaldehyde **2** (0.2 g, 0.99 mmol) in 1.38 mL of dimethylformamide was added ethyl chloroacetate **3** (0.12 g, 0.99 mmol) followed by the addition of K_2_CO_3_ (0.21 g, 1.49 mmol). Then, the obtained mixture was heated at 92–94 °C with continuous stirring for 4 h. TLC indicated the reaction’s completion, and the solution was added to ice water and the resulting precipitates were filtered. The precipitates were washed and recrystallized in methanol to obtain the pure product [50].

#### 3.1.2. Synthesis of 5-Bromobenzofuran-2-Carbohydrazide (**5**)

Ester **4** (0.2 g, 0.74 mmol) was dissolved in ethanol (30 mL) and the temperature dropped to 0 °C. Hydrazine monohydrate (0.15 mL, 2.97 mmol) was added and the resulting solution stirred overnight at 25 °C under TLC monitoring. After reaction completion (12 h), the solvent was evaporated to obtain the pure product [53].

#### 3.1.3. General Procedure for the Synthesis of 5-(5-Bromobenzofuran-2-yl)-4-phenyl-4*H*-1,2,4-triazole-3-thione (**8**)

The hydrazide **5** (0.2 g, 0.78 mmol) was dissolved in ethanol (15 mL) and diversely substituted phenyl isothiocyanate **6** (0.78 mmol) was added to it. The resulting contents were refluxed for 2 h. The formed intermediate **7** precipitates were filtered out, washed, and dried. The obtained thiosemicarbazide **7** was dissolved in a basic KOH (0.065 g, 1.17 mmol) solution in water (5 mL) and reflux for 4 h. After cooling, precipitates were formed by acidifying with dilute HCl, which were filtered off and recrystallized to provide the corresponding triazole **8** [26].

#### 3.1.4. General Synthetic Procedures for the Synthesis of 2-((5-(5-Bromobenzofuran-2-yl)-4-phenyl-4*H*-1,2,4-triazol-3-yl)thio)-N-phenylacetamide (**10a**–**e**)

Three methods for *S*-alkylation of 5-(5-bromobenzofuran-2-yl)-4-phenyl-4*H*-1,2,4-triazole-3-thione **8** to 2-((5-(5-bromobenzofuran-2-yl)-4-phenyl-4*H*-1,2,4-triazol-3-yl)thio)-*N*-phenylacetamide **10a**–**e** were employed:

*Method A. Conventional method*

A mixture of triazole **8** (1 equiv.) and LiH (1.2 equiv.) in DMF was stirred for half an hour. Then, the appropriate 2-bromo-*N*-arylacetamide (**9a**–**e**, 1 equiv.) was added to the resulting mixture and stirred at room temperature for 15–24 h. Upon reaction completion (indicated by TLC), petroleum ether was added to obtain the precipitates of the final product (**10a**–**e**). The obtained precipitates were filtered, washed with water, and dried at room temperature. The pure product was obtained by recrystallization with ethanol [54].

*Method B. Microwave-assisted method*

A solution containing 1,2,4-triazole-3-thione **8** (1 equiv.) and LiH (1.2 equiv.) in DMF was stirred for half an hour. Then the appropriate 2-bromo-*N*-arylacetamide (**9a**–**e**, 1 equiv.) was added to the resulting mixture, which was irradiated with microwaves in a microwave oven for 60 to 75 s. After reaction completion, petroleum ether was poured to obtain precipitates of the final product (**10a**–**e**). The formed precipitates were filtered, washed with water, and recrystallized with ethanol to obtain the pure product [54]. 

*Method C. Ultrasound-assisted method*

To a solution of triazole-3-thione **8** (1 equiv.) in ethanol (5 mL), triethylamine (1 equiv.) and corresponding 2-bromo-*N*-arylacetamide (**9a**–**e**, 1 equiv.) ware added. The resultant solution was sonicated at room temperature for 15–30 min in an ultrasonic cleaning bath. Upon reaction completion, excess ethanol was removed under vacuum in order to produce the crude product, which was further recrystallized from ethanol to obtain the pure product [55].

### 3.2. Characterization Data

*2-((5-(5-bromobenzofuran-2-yl)-4-phenyl-4H-1,2,4-triazol-3-yl)thio)-N-phenylacetamide* (**10a**): Off white powder, m.p. 230 °C; FT-IR (KBr, cm*^−^*^1^): *v* 3029 (N-H), 1598 (C=N), 1327 (C-N), 689 (C-S), 1674 (C=O), 1538 (C=C), 1242 (C-O-C), 1445 (-CH_2_); ^1^H-NMR, CDCl_3_, 400 MHz (δ/ppm): 4.07 (s, 2H, CH_2_), 6.38–7.99 (m, 14H, aryl-H/benzofuran-H), 10.29 (s, 1H, CONH); ^13^C-NMR, CDCl_3_, 100 MHz (δ/ppm): 31.60, 107.58, 113.39, 117.01, 119.93, 124.42, 124.61, 127.42, 129.05, 129.18, 129.62, 130.69, 131.58, 132.62, 138.34, 143.18, 147.80, 153.75, 155.01, 162.71, 166.11; MS (ESI) *m*/*z*: 507.05 [M + 1]^+^; Anal. Calcd. For C_24_H_17_BrN_4_O_2_S: C, 57.04; H, 3.39; N, 11.09; Found: C, 57.07; H, 3.41; N, 11.07.

*2-((5-(5-Bromobenzofuran-2-yl)-4-phenyl-4H-1,2,4-triazol-3-yl)thio)-N-(3,4-dimethylphenyl)acetamide* (**10b**): Gray powder, m.p. 227 °C; FT-IR (KBr, cm*^−^*^1^): *v* 3278 (N-H), 1548 (C=N), 1330 (C-N), 658 (C-S), 1679 (C=O), 1506 (C=C), 1207 (C-O-C), 1442 (-CH_2_); ^1^H-NMR, CDCl_3_, 400 MHz (δ/ppm): 2.17 (s, 6H, 2CH_3_), 4.01 (s, 2H, CH_2_), 6.35–7.59 (m, 12H, aryl-H/benzofuran-H), 10.01 (s, 1H, CONH); ^13^C-NMR, CDCl_3_, 100 MHz (δ/ppm): 19.35, 19.95, 36.41, 107.53, 113.38, 117.44, 118.51, 121.09, 124.59, 127.41, 128.27, 129.21, 129.59, 130.02, 130.67, 131.53, 133.33, 136.00, 137.28, 137.99, 143.29, 153.73, 159.53, 160.08, 167.80; MS (ESI) *m*/*z*: 535.04 [M + 1]^+^; Anal. Calcd. For C_26_H_21_BrN_4_O_2_S: C, 58.54; H, 3.97; N, 10.50; Found: C, 58.57; H, 3.98; N, 10.48.

*2-((5-(5-Bromobenzofuran-2-yl)-4-phenyl-4H-1,2,4-triazol-3-yl)thio)-N-(4-nitrophenyl)acetamide* (**10c**): Off white powder, m.p. 118–120 °C; FT-IR (KBr, cm*^−^*^1^): *v* 2987 (N-H), 1588 (C=N), 1313 (C-N), 676 (C-S), 1679 (C=O), 1512 (C=C), 1262 (C-O-C), 1444 (-CH_2_); ^1^H-NMR, CDCl_3_, 400 MHz (δ/ppm): 4.21 (s, 2H, CH_2_), 6.43–8.61 (m, 13H, aryl-H/benzofuran-H), 11.12 (s, 1H, CONH); ^13^C-NMR, CDCl_3_, 100 MHz (δ/ppm): 36.27, 107.00, 113.33, 116.28, 121.13, 123.11, 124.19, 124.44, 125.81, 127.56, 129.19, 130.51, 131.23, 135.72, 142.43, 143.91, 144.96, 156.33, 159.88, 160.47, 167.72; MS (ESI) *m*/*z*: 552.03 [M + 1]^+^; Anal. Calcd. For C_24_H_16_BrN_5_O_4_S: C, 52.37; H, 2.93; N, 12.72; Found: C, 52.35; H, 2.96; N, 12.74.

*2-((5-(5-Bromobenzofuran-2-yl)-4-(3,4-dichlorophenyl)-4H-1,2,4-triazol-3-yl)thio)-N-(2,5-dimethoxyphenyl)acetamide* (**10d**): Gray powder, m.p. 190 °C; FT-IR (KBr, cm*^−^*^1^): *v* 3087 (N-H), 1601 (C=N), 1317 (C-N), 670 (C-S), 1693 (C=O), 1551 (C=C), 1221 (C-O-C), 1474 (-CH_2_); ^1^H-NMR, CDCl_3_, 400 MHz (δ/ppm): 3.73 (s, 3H, OCH_3_), 3.85 (s, 3H, OCH_3_), 4.12 (s, 2H, CH_2_), 6.55–8.02 (m, 10H, aryl-H/benzofuran-H), 9.48 (s, 1H, CONH); ^13^C-NMR, CDCl_3_, 100 MHz (δ/ppm): 36.66, 55.94, 56.68, 106.47, 107.65, 109.18, 111.23, 113.34, 117.12, 124.64, 126.86, 128.43, 129.21, 129.47, 132.02, 132.16, 134.52, 134.55, 135.95, 142.90, 143.50, 153.88, 155.52, 157.91, 162.70, 165.79; MS (ESI) *m*/*z*: 634.97 [M + 1]^+^; Anal. Calcd. For C_26_H_19_BrCl_2_N_4_O_4_S: C, 49.23; H, 3.02; N, 8.83; Found: C, 49.20; H, 3.06; N, 8.80.

*2-((5-(5-Bromobenzofuran-2-yl)-4-(3,4-dichlorophenyl)-4H-1,2,4-triazol-3-yl)thio)-N-(3,4-dichlorophenyl)acetamide* (**10e**): Gray powder, m.p. 219 °C; FT-IR (KBr, cm*^−^*^1^): *v* 3090 (N-H), 1591 (C=N), 1378 (C-N), 671 (C-S), 1681 (C=O), 1540 (C=C), 1273 (C-O-C), 1474 (-CH_2_); ^1^H-NMR, CDCl_3_, 400 MHz (δ/ppm): 4.00 (s, 2H, CH_2_), 6.67–7.99 (m, 10H, aryl-H/benzofuran-H), 10.32 (s, 1H, CONH); ^13^C-NMR, CDCl_3_, 100 MHz (δ/ppm): 36.51, 107.92, 113.38, 117.27, 119.22, 121.51, 124.75, 126.76, 127.64, 129.06, 129.41, 129.82, 130.56, 131.81, 132.25, 132.87, 134.84, 136.39, 137.69, 142.96, 153.83, 156.82, 163.80, 166.25; MS (ESI) *m*/*z*: 642.88 [M + 1]^+^; Anal. Calcd. For C_24_H_13_BrCl_4_N_4_O_2_S: C, 44.82; H, 2.04; N, 8.71; Found: C, 44.84; H, 2.02; N, 8.69.

### 3.3. AChE Inhibition Assay

The inhibitory effects of all synthesized derivatives on AChE activities were measured using a modified Ellman’s method [56,57]. This experimental study used DTNB (5,5′-dithio-bis(2-nitro-benzoic)acid) and AChI as the substrate and chromogen. The tested compounds (10 µL; 10–100 Mm) were mixed with AChE (50 µL; 38 U) solution and incubated for 10 min at 25 °C. DTNB (50 µL; 0.3 mM) was then added. The transfer of AChI (200 µL; 50 mM Tris/HCl buffer pH = 8) initiated the reaction. A spectrophotometer recorded the hydrolysis of AChI by the formation of yellow 5-thio-2-nitrobenzoate anions as a result of the reaction of DTNB with thiocholine at a wavelength of 412 nm. As a positive control, eserine (0.5 mM) was employed. The given formula was used to calculate the (%) inhibition:

$$\mathrm{Inhibition}\;\left(\%\right)=\frac{\mathrm{Control}-\mathrm{Test}}{\mathrm{Control}}\times 100$$

IC_50_ values were calculated using data from GraphPad Prism 5 software.

### 3.4. Antibacterial Assay

The antibacterial potential of all targeted compounds was evaluated using the disc diffusion method [58,59]. A suspension of 100 μL containing 10^8^ cfu/mL of bacteria was applied on nutrient agar media with the support of a sterilized loop. The compound solution was infused into sterilized filter paper discs (5.6 mm in diameter) at a particular concentration (25 μg/100 μL) in chloroform. The standard penicillin (25 μg per disc) was utilized as a positive control for antibacterial assay. These discs were carefully placed on agar which had originally been immunized with selected bacterial cells. The agar culture plates were stored at 4 °C for 1 h and then the bacterial culture plate was incubated at 27 °C for 24 h. The inhibition zone was determined in millimeters (including the 5.6 mm disc diameter) with a reference control.

### 3.5. In Silico Modeling Method

The synthesized derivatives were further in silico modeled to examine their anticholinesterase activity using the Induced Fit docking (IFD) module of Molecular Operating Environment 2015.10 (MOE 2015). The three-dimensional (3D) X-ray crystalized structure of acetylcholinesterase (PDB ID: 4EY6; 2.4 Å) was obtained from Protein Data Bank (PDB) (https://www.rcsb.org/). The 3D conformer of galantamine (CID: 9651) was fetched from the PubChem database, and served as the standard for the acetylcholinesterase inhibitor. The compound **10d** was sketched in BIOVIA Discovery Studio v16.10, and its energy was minimized by the DREIDING forcefield. The Structural Preparation tool of MOE was used to prepare the macromolecule to inspect and fix structural issues such as protonation state, missing residues, termini capping, and alternates. The extraneous co-factor and unbound water molecules were eliminated. The AChE structure was further protonated and optimized using the Protonate 3D tool of MOE, and the energy of the biomolecular system was minimized with the Amber10:EHT forcefield. The binding pocket was identified in the vicinity of the co-crystalized native inhibitor and the triangular matcher placement method was used to dock the test compounds and the London dG was used to score them. The docked poses were further refined using the Induced Fit method, and subsequent poses were scored with the GBVI/WSA dG scoring feature. The docking methods were validated by cognate redocking of the native ligand with RMSD as a validating measure. The pose with the lowest binding energy (i.e., S) was utilized to further simulate the ligand–receptor interactions using Discovery Studio Visualizer v16.10.

## 4. Conclusions

In this article, novel benzofuran derivatives **10a**–**e** containing a 1,2,4-triazole moiety were designed, synthesized, and subjected to pharmacological evaluation. The results demonstrated that most of the benzofuran-triazole derivatives exhibited moderate to good inhibitory potential against AChE. Among all compounds, **10d** bearing the 2,5-dimethoxyphenyl moiety possessed high potency against AChE with an IC_50_ value of 0.55 ± 1.00 µM. Antibacterial activity results clearly identified compound **10b** with 3,4-dimethyl substituents as the most potent antibacterial agent against *B. subtilis*, with a MIC value of 1.25 μg/mL, which is comparable to that of penicillin. Compound **10a** possessing unsubstituted phenyl rings, with an IC_50_ value of 1.80 ± 0.25 µM, also had promising antibacterial potential against *E. coli*. The structure–activity relationship revealed that AChE inhibitory activity of the synthesized derivatives was influenced by the nature of the substituent attached to the phenyl ring of acetamide and triazole. IFD simulation further complemented experimental AChE inhibition by delineating the potential mechanism of action for AChE inhibition, and highlighted **10d** as a potential lead candidate to develop novel benzofuran-based anticholinesterase drugs in therapeutics of Alzheimer’s disease. Overall, the current research suggests that further modifications in benzofuran-based triazole derivatives may give rise to more efficacious anti-AChE and antibacterial compounds.

## Figures and Tables

**Table 1:** Comparative study of conventional, microwave- and ultrasound-assisted methods.

Compounds	Reaction Yield			Reaction Time		
	Conventional (%)	Microwave (%)	Ultrasound (%)	Conventional (h)	Microwave (s)	Ultrasound (min)
** 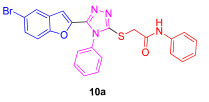 **	47	85	69	18	69	25
** 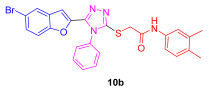 **	56	90	77	22	75	30
** 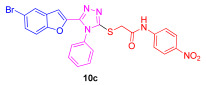 **	49	88	67	15	63	20
** 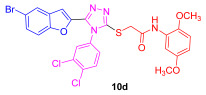 **	52	93	78	24	72	15
** 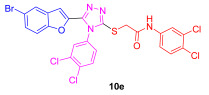 **	55	80	73	20	60	22

**Table 2:** Inhibitory effect of benzofuran-based 1,2,4-triazole derivatives **10a**–**e** on the activity.

Compounds	Inhibition % (0.5 mM)	IC_50_ (µM)
**10a**	65.84 ± 1.50	0.88 ± 0.50
**10b**	61.7 ± 1.50	1.50 ± 1.00
**10c**	55.90 ± 1.25	2.28 ± 1.75
**10d**	81.45 ± 0.50	0.55 ± 1.00
**10e**	51.23 ± 1.24	1.98 ± 0.25
Eserine	93.20 ± 1.15	0.06 ± 0.01

**Table 3:** Antibacterial evaluation by measuring ZI in millimeters.

Compounds	*B. subtilis*		*E. coli*	
	Inhibition Zone (mm)	MIC (µg/mL)	Inhibition Zone (mm)	MIC (µg/mL)
** 10a **	Nd	Nd	38 ± 1.50	1.80 ± 0.25
** 10b **	42 ± 1.00	1.25 ± 0.60	11.5 ± 1.40	3.8 ± 2.1
** 10c **	nd *	nd *	28 ± 0.00	2.5 ± 0.70
** 10d **	11 ± 0.50	9.25 ± 1.25	12 ± 0.65	16 ± 1.00
** 10e **	8 ± 0.75	14 ± 1.80	24 ± 0.50	2.5 ± 1.40
Penicillin	18 ± 0.50	1 ± 1.50	24 ± 0.50	2.4 ± 1.00

* Not Determined.

**Table 4:** IFD parameters for ligands docked in the AChE active site.

Ligand	Binding Score (S) Kcal/mol	Interacting Residues	Interaction Type
Galantamine	−7.07	TRP86, GLY121, TYR337, GLU202, SER125, GLY120, ASN87	H-bonding, Amide-π stacked, π-Alkyl
**10d**	−9.34	HIS447, TRP86, GLY121, GLY120, TYR133, TYR341, PHE338, VAL294, PHE297, TRP286, TYR124, TYR337	H-bonding, π-sulfur, π-π stacked, π-π T-shaped, alkyl, π-alkyl

## Data Availability

Not applicable.

## Supplementary Materials

The following supporting information can be downloaded at: https://www.mdpi.com/article/10.3390/molecules28166007/s1, Figures S1–S10: 1H and 13C NMR spectra of compound 10a–e.; Figure S11: Cognate re-docking of co-crystalized ligand of AChE (4EY6) to validate IFD protocol; Co-crystalized apo-conformation (green) of native ligand aligned against its re-docked conformation reproduced by IFD protocol.
