# Supplementary material for: Unfolding the Antibacterial Activity and Acetylcholinesterase Inhibition Potential of Benzofuran-Triazole Hybrids: Synthesis, Antibacterial, Acetylcholinesterase Inhibition, and Molecular Docking Studies
Source: Molecules. 2023 Aug 10;28(16):6007. doi: 10.3390/molecules28166007 (PMC10459521; doi:10.3390/molecules28166007)
Supplement: Supplementary file 1 [file molecules-28-06007-s001.zip › molecules-2467749-supplementary.pdf]

Supporting information for

Unfolding the Antibacterial Activity and Acetylcholinesterase Inhibition Potential of Benzofuran-Triazole Hybrids: Synthesis, Antibacterial, Acetylcholinesterase Inhibition, and Molecular Docking Studies

Sadaf Saeed[1], Ameer Fawad Zahoor[1]*, Shagufta Kamal[2], Zohaib Raza[3], Mashooq Ahmad Bhat [4]**

1 Department of Chemistry, Government College University Faisalabad, Faisalabad-38000, Pakistan. sadafsaeed932@gmail.com; fawad.zahoor@gcuf.edu.pk

2 Department of Biochemistry, Government College University Faisalabad, Faisalabad 38000, Pakistan. shaguftakamal81@gmail.com

3 Department of Chemistry, School of Physical Sciences, university of Adelaide, Adelaide, S A 5000, Australia. zohaib.raza@adelaide.edu.au

4 Department of Pharmaceutical Chemistry, College of Pharmacy, King Saud University, Riyadh 11451, Saudi Arabia. mabhat@ksu.edu.sa

* Correspondence: Ameer Fawad Zahoor; fawad.zahoor@gcuf.edu.pk; Mashooq Ahmad Bhat; mabhat@ksu.edu.sa

^{1}H and ^{13}C NMR spectra of compound **10a-e**

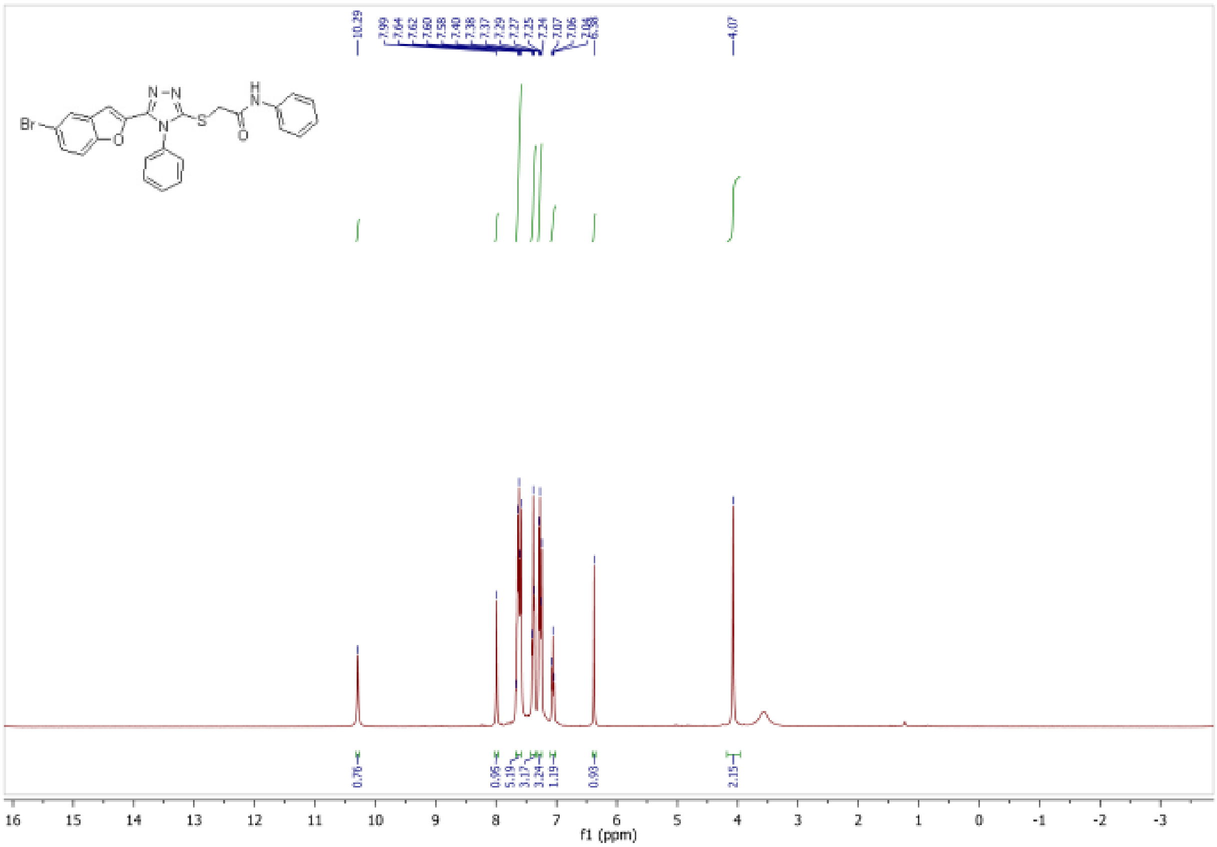

Figure S1: ^{1}H NMR spectrum of 2-((5-(5-bromobenzofuran-2-yl)-4-phenyl-4*H*-1,2,4-triazol-3-yl)thio)-*N*-phenylacetamide (**10a**).

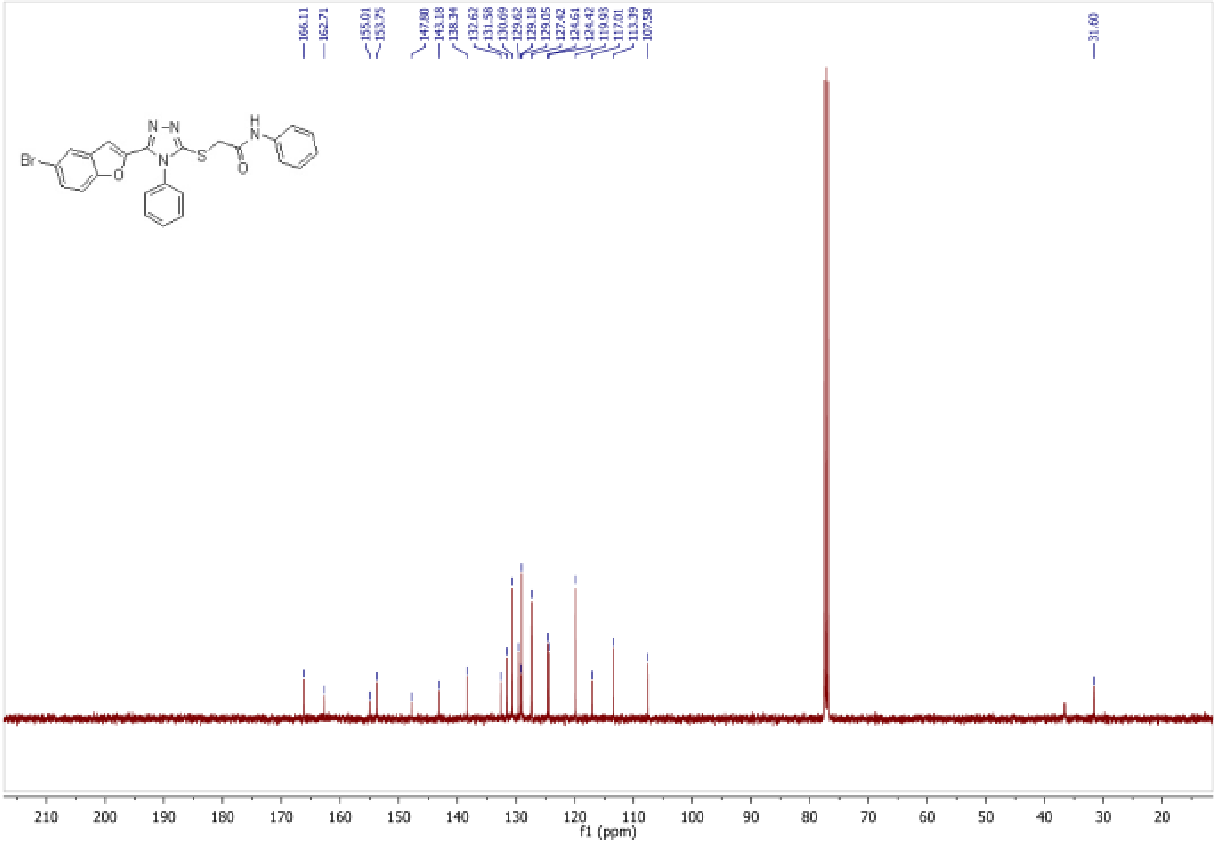

Figure S2: ^{13}C NMR spectrum of 2-((5-(5-bromobenzofuran-2-yl)-4-phenyl-4*H*-1,2,4-triazol-3-yl)thio)-*N*-phenylacetamide (**10a**).

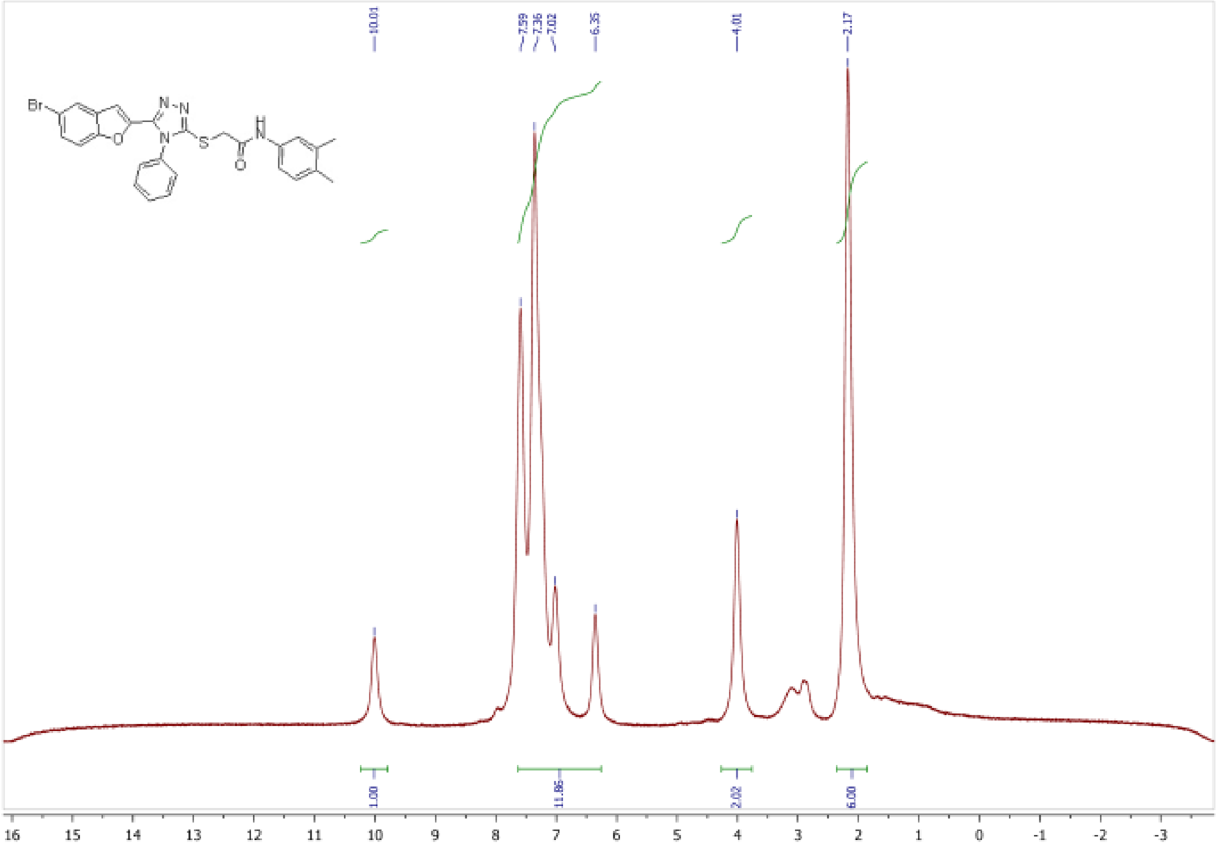

Figure S3: ^{1}H NMR spectrum of 2-((5-(5-bromobenzofuran-2-yl)-4-phenyl-4*H*-1,2,4-triazol-3-yl)thio)-*N*-(3,4-dimethylphenyl)acetamide (**10b**).

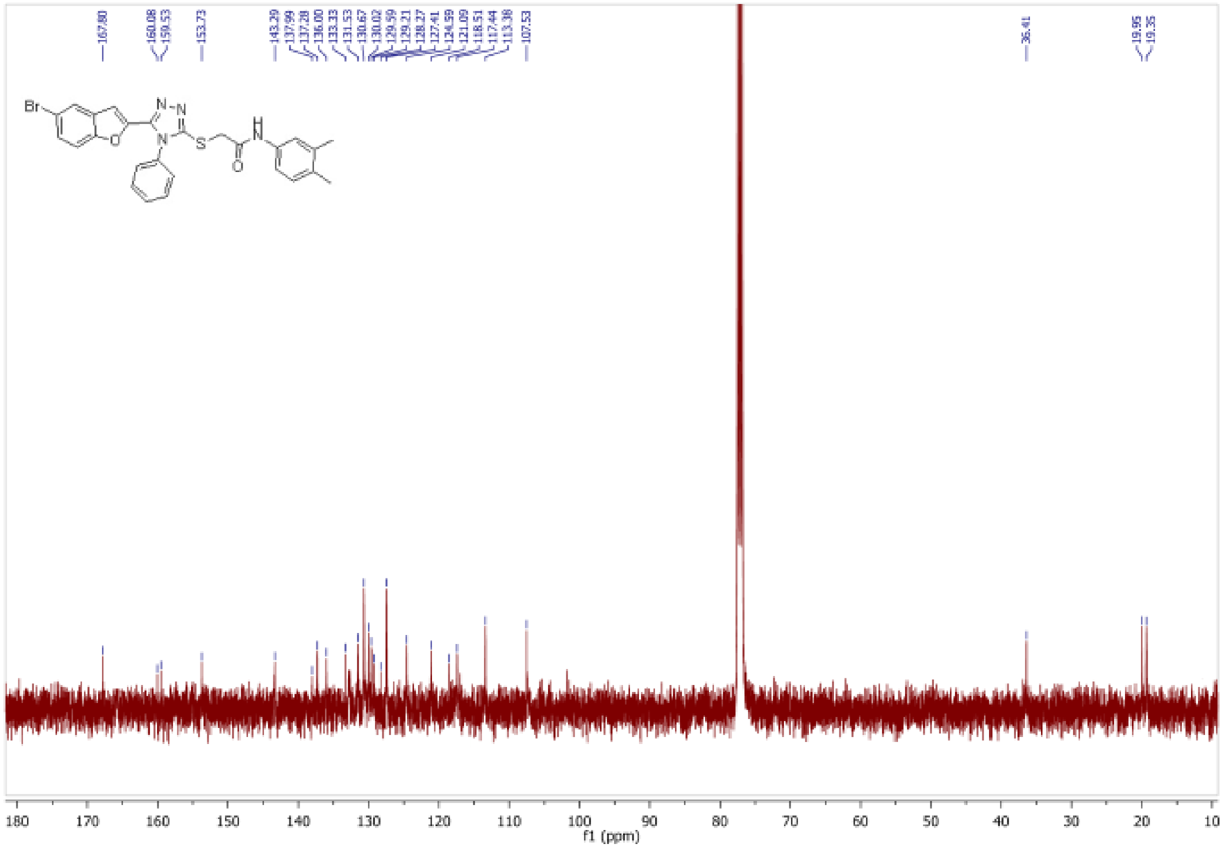

Figure S4: ^{13}C NMR spectrum of 2-((5-(5-bromobenzofuran-2-yl)-4-phenyl-4*H*-1,2,4-triazol-3-yl)thio)-*N*-(3,4-dimethylphenyl)acetamide (**10b**).

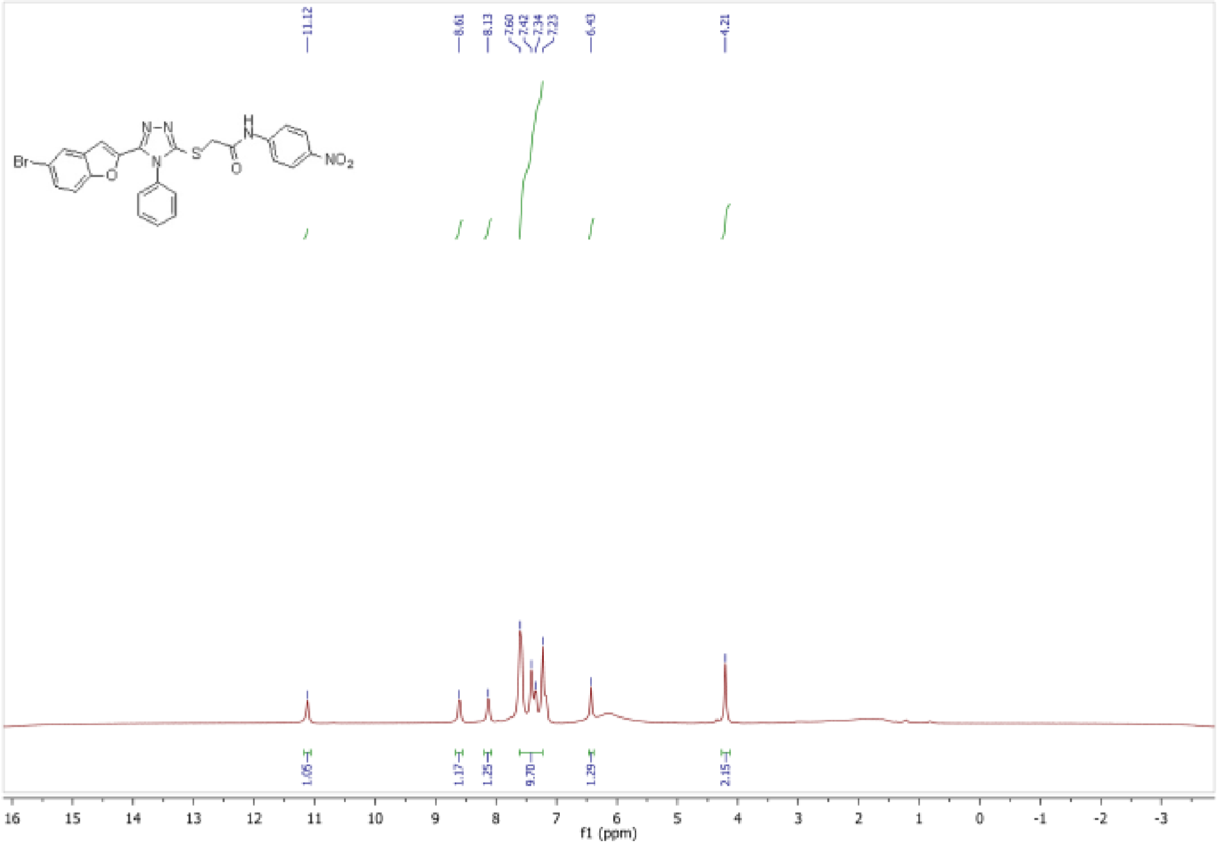

Figure S5: ^{1}H NMR spectrum of 2-((5-(5-bromobenzofuran-2-yl)-4-phenyl-4*H*-1,2,4-triazol-3-yl)thio)-*N*-(4-nitrophenyl)acetamide (**10c**).

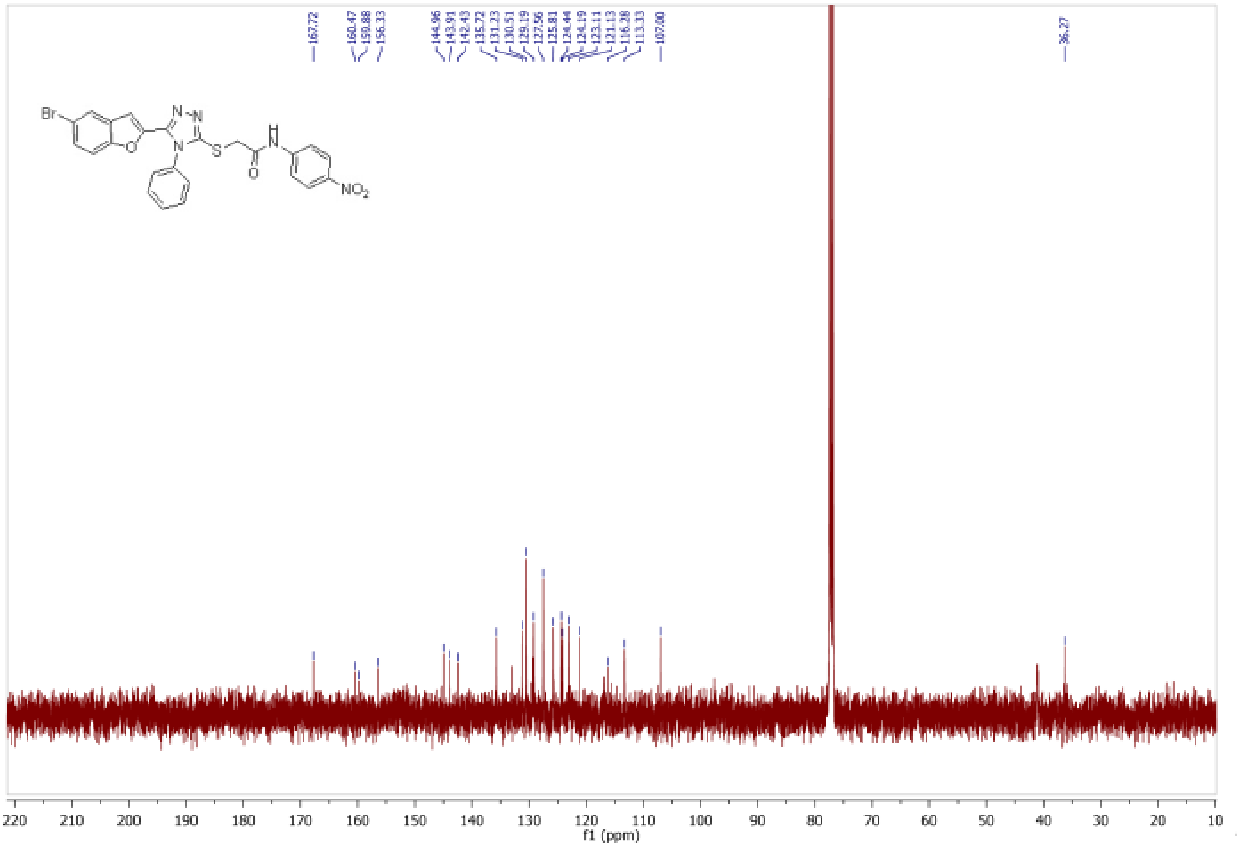

Figure S6: ^{13}C NMR spectrum of 2-((5-(5-bromobenzofuran-2-yl)-4-phenyl-4*H*-1,2,4-triazol-3-yl)thio)-*N*-(4-nitrophenyl)acetamide (**10c**).

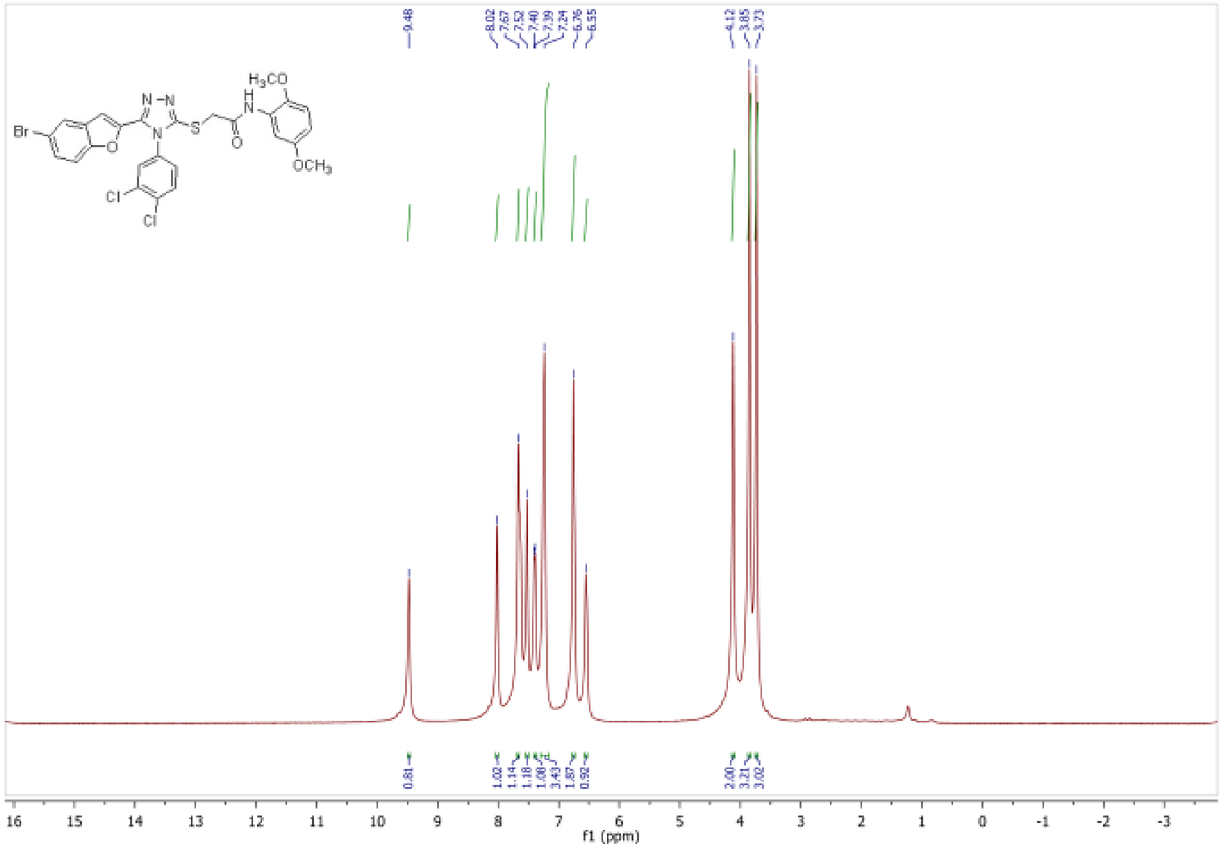

Figure S7: ^{1}H NMR spectrum of 2-((5-(5-bromobenzofuran-2-yl)-4-(3,4-dichlorophenyl)-4*H*-1,2,4-triazol-3-yl)thio)-*N*-(2,5-dimethoxyphenyl)acetamide (**10d**).

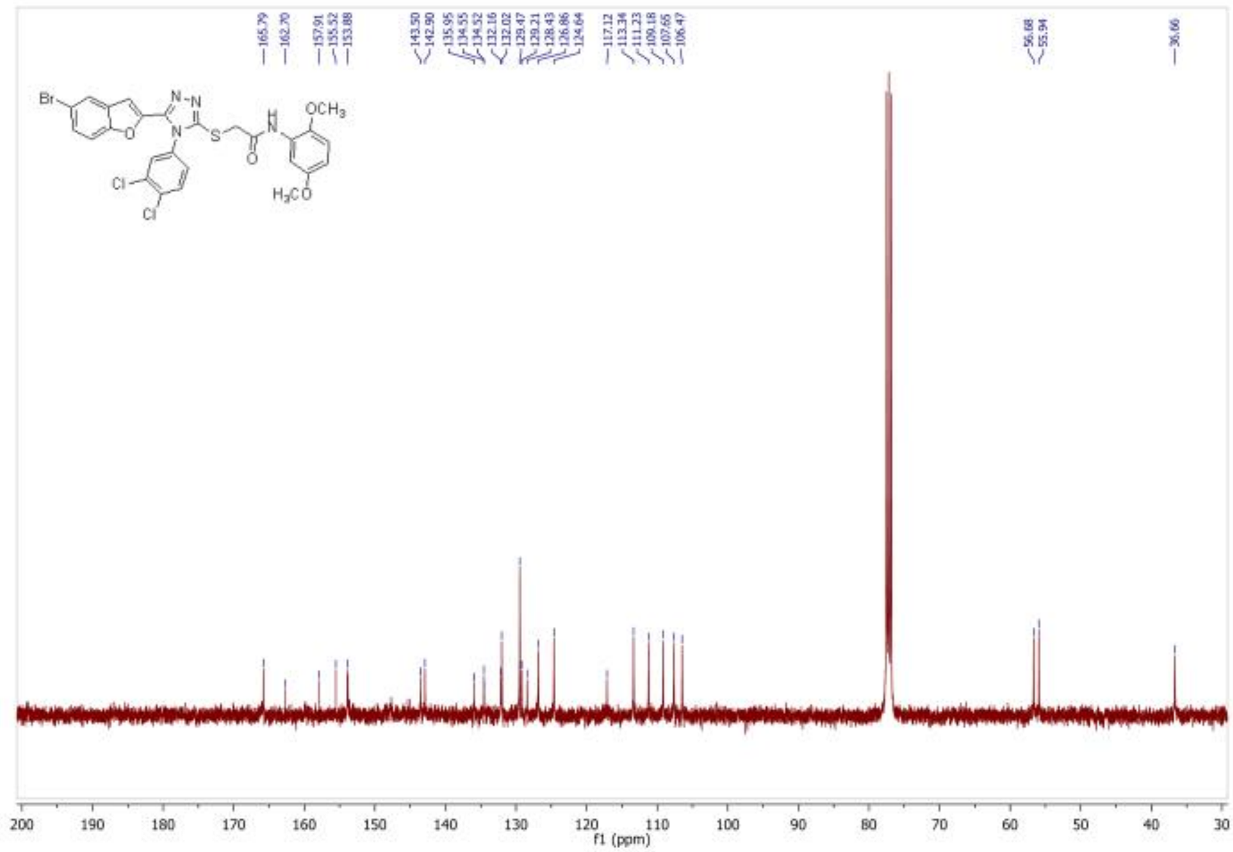

Figure S8: ^{13}C NMR spectrum of 2-((5-(5-bromobenzofuran-2-yl)-4-(3,4-dichlorophenyl)-4*H*-1,2,4-triazol-3-yl)thio)-*N*-(2,5-dimethoxyphenyl)acetamide (**10d**).

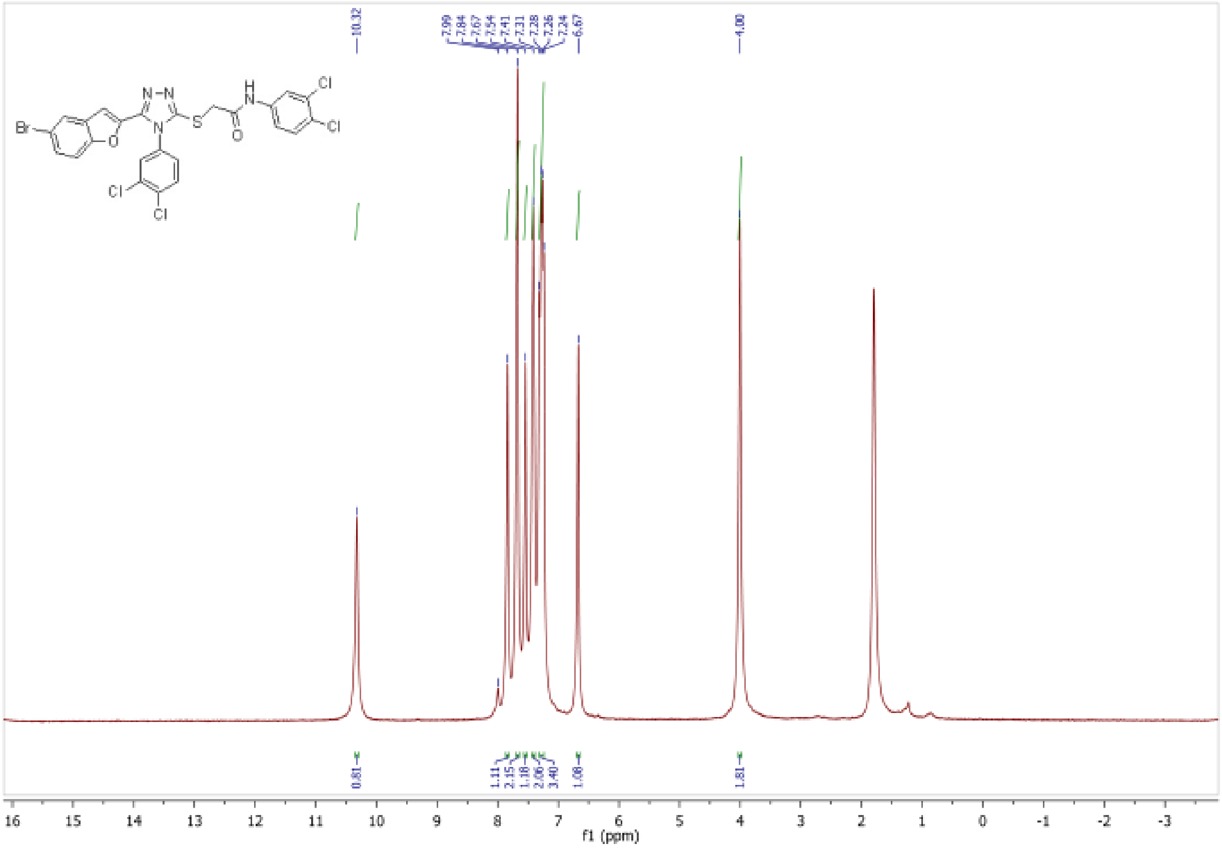

Figure S9: ^{1}H NMR spectrum of 2-((5-(5-bromobenzofuran-2-yl)-4-(3,4-dichlorophenyl)-4*H*-1,2,4-triazol-3-yl)thio)-*N*-(3,4-dichlorophenyl)acetamide (**10e**).

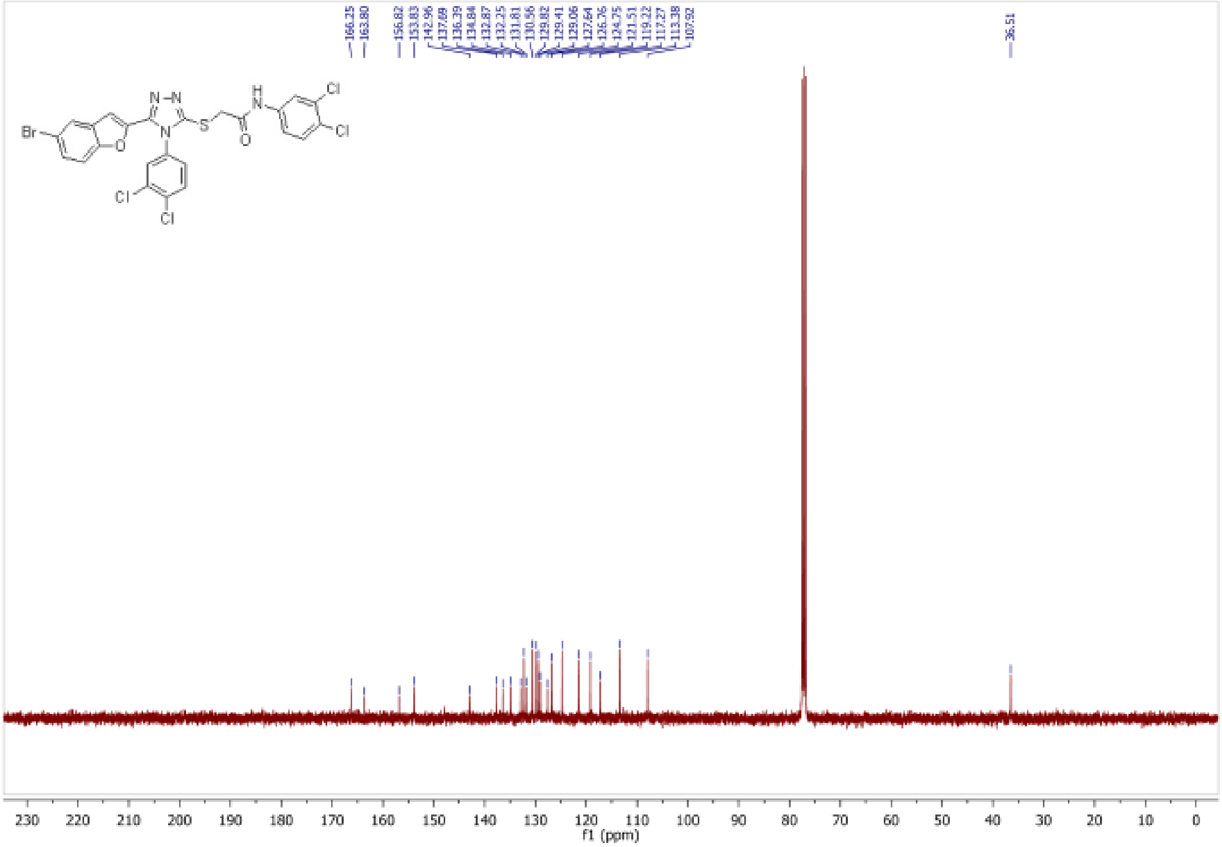

Figure S10: ^{13}C NMR spectrum of 2-((5-(5-bromobenzofuran-2-yl)-4-(3,4-dichlorophenyl)-4*H*-1,2,4-triazol-3-yl)thio)-*N*-(3,4-dichlorophenyl)acetamide (**10e**).

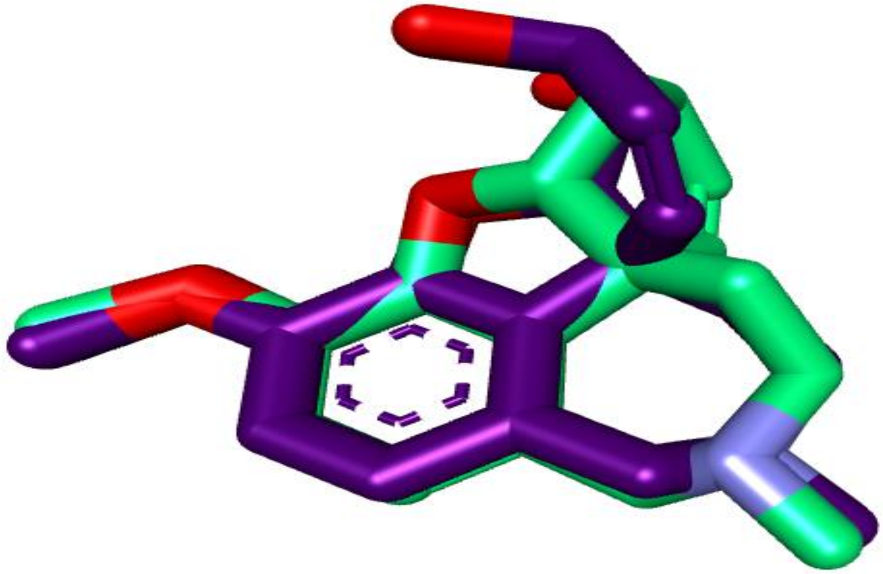

Figure S11. Cognate re-docking of co-crystalized ligand of AChE (4EY6) to validate IFD protocol; Co-crystalized apo-conformation (green) of native ligand aligned against its re-docked conformation reproduced by IFD protocol.
